# Transition Metal Layer Substitution in Mo_2_CS_2_ MXene for Improving Li Ion Surface Kinetics

**DOI:** 10.1021/acsomega.3c02080

**Published:** 2023-06-14

**Authors:** Konstantina A. Papadopoulou, Stavros-Richard G. Christopoulos

**Affiliations:** † Department of Physics and Astronomy, Faculty of Environment, Science and Economy, University of Exeter, Exeter EX4 4QL, U.K.; ‡ Faculty of Engineering, Environment and Computing, Coventry University, Priory Street, Coventry CV1 5FB, U.K.; ¶ Department of Computer Science, School of Computing and Engineering, University of Huddersfield, Huddersfield HD4 6DJ, U.K.; § Centre for Computational Science and Mathematical Modelling, Coventry University, Coventry CV1 2TU, U.K.

## Abstract

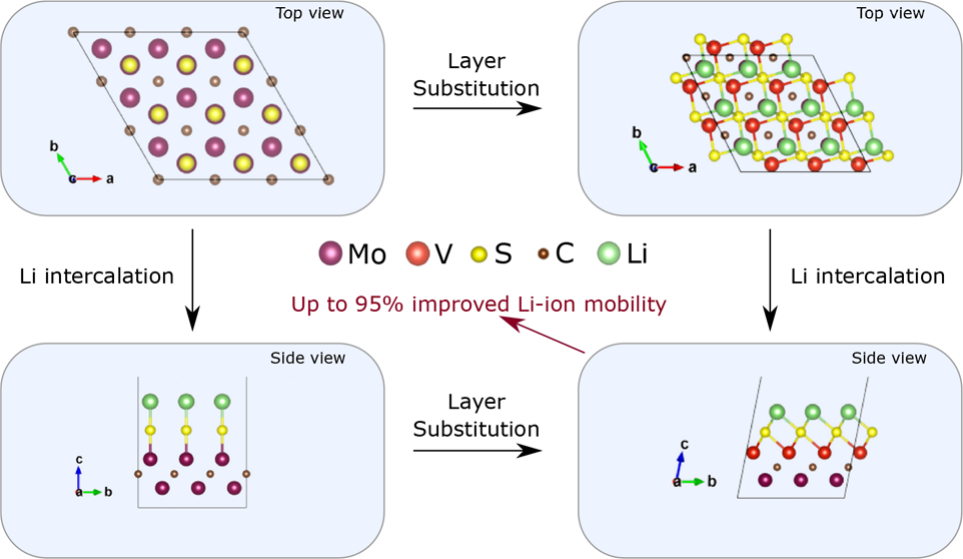

We study the adsorption and mobility of a Li ion on the surface of the Mo_2_CS_2_ MXene by means of Density Functional Theory. We find that by substituting the Mo atoms of the upper MXene layer with V the mobility of the Li ion can be improved up to 95% while the material retains its metallic character. This fact indicates that MoVCS_2_ is a promising candidate for anode electrode in Li-ion batteries, where the materials need to be conductive and the Li ion needs to have a small migration barrier.

## Introduction

The tunable properties^1−5^ via control of the surface termination atoms of two-dimensional (2D) materials has made the latter the new point of interest regarding their use as anode materials in Li-ion batteries. Since 2011, a new ceramic material belongs in the 2D class, the MXenes. MXenes have the general formula M_*n*+1_X_*n*_T_*x*_, (*n* = 1, 2, 3), with T a surface termination atom^2^ and M an early transition metal. X stands for either carbon (C) or nitrogen (N).^4,6−9^

The most studied MXene is titanium carbide, Ti_3_C_2_,^10−17^ which was also the first to be discovered^4,18^ in 2011. Since then, zirconium carbide, Zr_2_C, has garnered attention^5,19−22^ after it was experimentally synthesized^20^ by etching Al_3_C_3_ from layered Zr_3_Al_3_C_5_ structures. However, the studies of Zr_2_C as an anode electrode in ion batteries have been limited to O, OH, F, and S surface termination atoms, while a more extensive study of its use as an anode electrode in Li and non-Li ion batteries was conducted by Papadopoulou et al. in 2022.^5^

A year earlier than the synthesis of Zr_2_C, in 2015, another MXene, molybdenum carbide, Mo_2_C, was experimentally synthesized from the gallium-based atomic laminate Mo_2_Ga_2_C.^23^ Band structure calculations have showed that Mo_2_C is metallic when it is terminated by O or OH atoms, while it becomes semiconducting when it is terminated by F or Cl atoms.^23^ The need to find new electrode materials with large ion capacities and fast charging rates has led to the question of whether Mo_2_C can be used to enhance electrode performance.

In 2016, Sun et al.^24^ theoretically verified that the bare and terminated Mo_2_C is dynamically stable, a crucial property for an electrode. Also in 2016, Çakır et al.^25^ theoretically studied the adsorption and diffusion of Li, Na, K, and Ca atoms on the surface of the Mo_2_C MXene. They predicted that all the aforementioned atoms are strongly adsorbed on the surface of the monolayer MXene and found energy barriers for diffusions as low as 15 meV (K case).

In 2017, Mehta et al.^26^ studied the adsorption of a Li ion on the Mo_2_C monolayer doped with N and Mn using first-principles calculations. In particular, they (a) substituted one Mo atom from the upper MXene layer with Mn and (b) substituted a C atom with N. They found that the presence of the dopant atoms increases the Li adsorption and, thus, the ion storage capacity. However, there was no investigation regarding the mobility of the Li ion.

Finally, Mehta et al.^27^ in 2019 performed first-principles calculations in S-terminated Mo_2_C. They found that the Li ion has a diffusion barrier equal to 0.22 eV.

In the present work, we study a Li ion’s adsorption and diffusion in the Mo_2_CS_2_ structure where the upper Mo MXene layer has been entirely substituted by vanadium, V. We denote this structure as MoVCS_2_. We chose the S-terminated Mo_2_CS_2_ because previous studies^5,28,29^ have shown that S lowers the migration barrier of the Li ion. The significance of this study and the findings therein are 2-fold: First, we identify that the transition layer substitution results in a more stable material than the original configuration, a fact that opens the way for experimental work on MXenes with two different transition metals. Second, the very low Li-ion diffusion barriers calculated herein indicate the suitability of the aforementioned kind of MXenes for electrodes in Li-ion batteries.

## Computational Methods

For the bulk structure of the bare Mo_2_C, we used a starting structure typical of that found in theoretical analysis.^30^ We performed Density Functional Theory (DFT) calculations as implemented in the CASTEP package^31−35^ to derive the Mo_2_CS_2_ structure, where we randomly placed the S atom on top of the Mo atom, at a distance smaller than the bond length *R*_0_(39) between the S and Mo which is equal to 2.33 Å.^40^ DFT uses pseudopotentials^36^ generated on the fly for the electron–ion interactions.^37^ The exchange-correlation interactions between electrons were described using the corrected density functional of Perdew, Burke, and Ernzerhof (PBE) within the generalized gradient approximation (GGA).

A vacuum space of 30 Å was introduced to minimize the mirror interactions between adjacent MXene layers. A plane wave cutoff energy *E*_cut_ = 650 eV and a k-point spacing of 7 × 7 × 1 was used to converge the overall energy per formula unit to 0.5 × 10^–5^ eV. The structures were optimized using the BFGS geometry optimization method^35,38^ and relaxed until the residual forces on the atoms were less than 0.01 eV × Å^–1^. DFT produced a final structure where the interlayer distance between S and Mo increased by 28.3% from the one we had originally considered, while the interlayer distance between the two Mo layers decreased by 1.4%.

For the structures including the Li-ion, DFT was applied again after we inserted a single ion on the surface of Mo_2_CS_2_ at a distance from the S atom smaller than the bond length *R*_0_(39) between the S and Li which is equal to 1.46 Å.^40^

For the adsorption energy, *E*_ads_, for the Li ion on the Mo_2_CS_2_ surface, we used the following equation
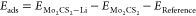
1where *E*_Reference_ is the total energy of a single Li atom in the metal phase, *E*_Mo_2_CS_2_–Li_ is the total energy of the structure including Li, and *E*_Mo_2_CS_2__ is the total energy of Mo_2_CS_2_ without the added ion.

Respectively, for the adsorption energy for the Li ion on the MoVCS_2_ surface, we used the following equation
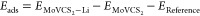
2where *E*_MoVCS_2_–Li_ is the total energy of the structure where the upper Mo layer has been substituted by V including the Li ion and *E*_MoVCS_2__ is the total energy of MoVCS_2_ without the added ion.

In order to calculate the energy barrier for diffusion, *E*_bar_, for the Li ion on each structure’s surface, first we created a 2 × 1 × 1 supercell of each structure. The initial (reactant) position of the Li ion was the one predicted by the DFT calculations, while the final (product) position was the same position in the adjacent cell. We then applied an LST/QST transition state (TS) search algorithm in CASTEP.^41^ The *E*_bar_ was calculated as the barrier from the initial position.

Finally, in order to calculate the Density of States (DOS) and thus characterize each material regarding its conductivity, we used the OPTADOS code^42^ as implemented in CASTEP. For convenience, the Fermi level was shifted at 0 eV.

## Results and Discussion

**Figure 1:**
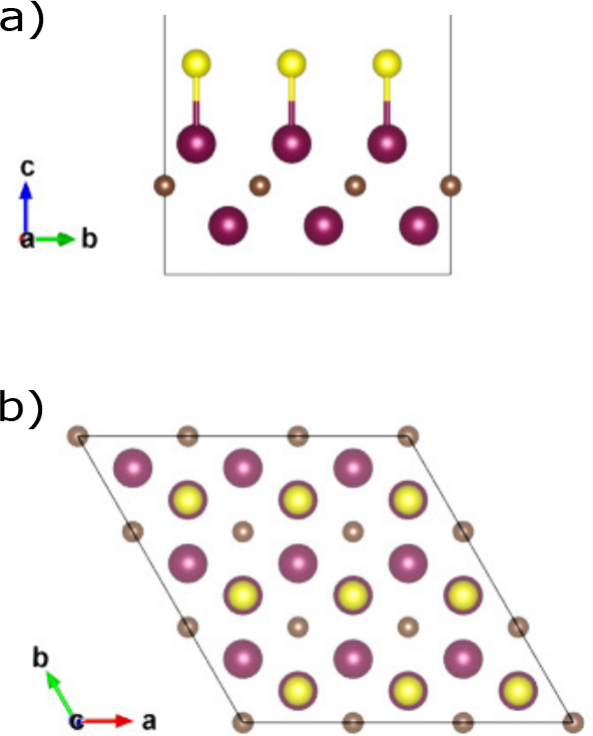
3 × 3 × 1 supercell for the Mo_2_CS_2_ MXene layer after geometry optimization: (a) front view, (b) top view. Purple spheres: Mo atoms. Brown spheres: C atoms. Yellow spheres: S atoms.

**Figure 2:**
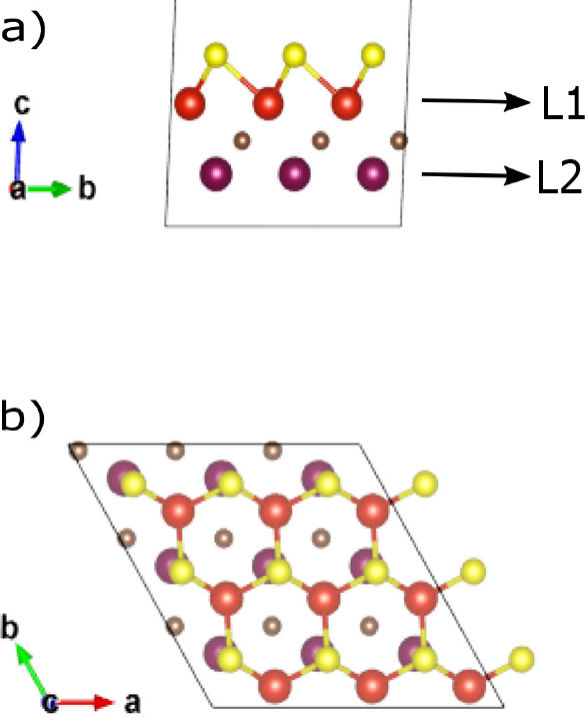
3 × 3 × 1 supercell for the MoVCS_2_ MXene layer after geometry optimization: (a) front view, (b) top view. Purple spheres: Mo atoms. Orange spheres: V atoms. Brown spheres: C atoms. Yellow spheres: S atoms.

The structure of the Mo_2_CS_2_ MXene is shown in Figure 1. The S atom sits directly above the Mo atom of the upper MXene layer. Furthermore, in Figure 2 we can see the structure of the MoVCS_2_ where the upper Mo layer of Mo_2_CS_2_ has been substituted by V. This time, the termination atom S sits above a bottom layer Mo atom. The upper and bottom MXene layers are denoted as L1 and L2 in Figure 2.

The coordination number of the S atom is also different. In Mo_2_CS_2_ S bonds with only one atom belonging in L1, and in MoVCS_2_ S bonds with three L1 atoms. Finally, the MoVCS_2_ structure has 3.25% lower final energy than the Mo_2_CS_2_ one, a fact that indicates that it is also stable.

**Figure 3:**
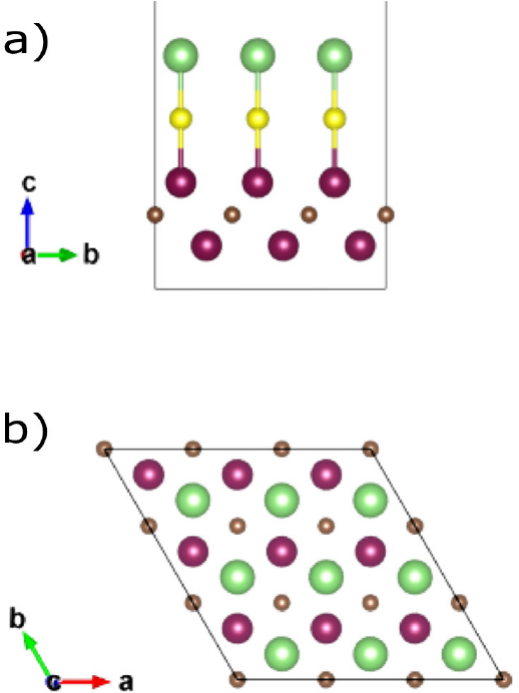
3 × 3 × 1 supercell for the Mo_2_CS_2_ MXene layer after geometry optimization: (a) front view, (b) top view. Purple spheres: Mo atoms. Brown spheres: C atoms. Yellow spheres: S atoms. Green spheres: Li ions.

**Figure 4:**
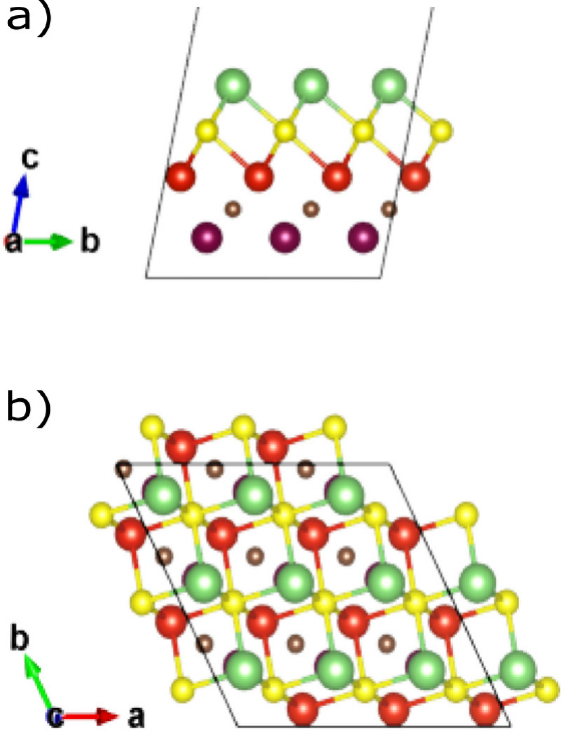
3 × 3 × 1 supercell for the MoVCS_2_ MXene layer after geometry optimization: (a) front view, (b) top view. Purple spheres: Mo atoms. Orange spheres: V atoms. Brown spheres: C atoms. Yellow spheres: S atoms. Green spheres: Li ions.

As a next step, we added the Li ion on the surface of the two structures. In Figure 3 we see the Mo_2_CS_2_ case where the Li ion sits on top of the S termination, while in Figure 4 we see the MoVCS_2_ case where the Li ion sits on top of a Mo atom of L2. In the latter case, the S termination atoms have slightly moved due to the bonds with Li and now sit above empty space. The MoVCS_2_–Li structure also has 3.1% lower final energy than the Mo_2_CS_2_–Li one, a fact that indicates its stability.

The adsorption energies for the Li ion on each structure’s surface are shown in Table 1. On Mo_2_CS_2_, Li appears to be more strongly adsorbed despite its smaller coordination number as described above. As Papadopoulou et al.^9^ have showed, *E*_ads_ depends on the average distance between the termination atoms, the Li ions, and the L1 atoms. In fact, the smaller these distances are, the stronger the adsorption. Our results here are fully in support of this observation, as the distances between the aforementioned atoms in Mo_2_CS_2_ are smaller, on average, than in MoVCS_2_ as indicated in Table 2.

**Table 1:** Ion Adsorption Energies *E*_ads_ and the Migration Barrier Energies *E*_bar_

Structure	*E*_ads_ (eV)	*E*_bar_ (eV)
Mo_2_CS_2_	–0.875	0.59
MoVCS_2_	–0.784	0.03

**Table 2:** Average Distances between Atoms in Mo_2_CS_2_ (L1 = Mo) and MoVCS_2_ (L1 = V)

Atoms	Mo_2_CS_2_ (Å)	MoVCS_2_ (Å)
L1–S	2.235	2.405
S–Li	2.205	2.387

Also in Table 1, we can see the migration barriers *E*_bar_ for the Li ion. One can see that the substitution of Mo with V lowers the energy barrier by 95% (0.59 eV in Mo_2_CS_2_ vs 0.03 eV in MoVCS_2_). This fact is significant since we seek to improve the mobility of the Li ion in order to achieve faster charge/discharge rates in Li-ion batteries.

Mehta et al.^27^ cite a migration barrier for Li in Mo_2_CS_2_ equal to 0.22 eV. This difference with the results reported here has to do with the different structure reported for Mo_2_CS_2_–Li where Mehta et al. found that S sits on top of a C atom. This difference is a result of the different softwares used to perform DFT (VASP vs CASTEP), as well as the different pseudopotentials used. Still, if we were to accept that *E*_bar_ = 0.22 eV, MoVCS_2_ exhibits 86.4% lower Li diffusion barrier. This reduction is also of high significance.

This result of *E*_bar_ = 0.03 eV we found here for MoVCS_2_ is equal with the one found for the Ti_3_C_2_Cl_2_ MXene in a previous study,^9^ further reinforcing the use of MXenes as anode electrodes in Li-ion batteries.

**Figure 5:**
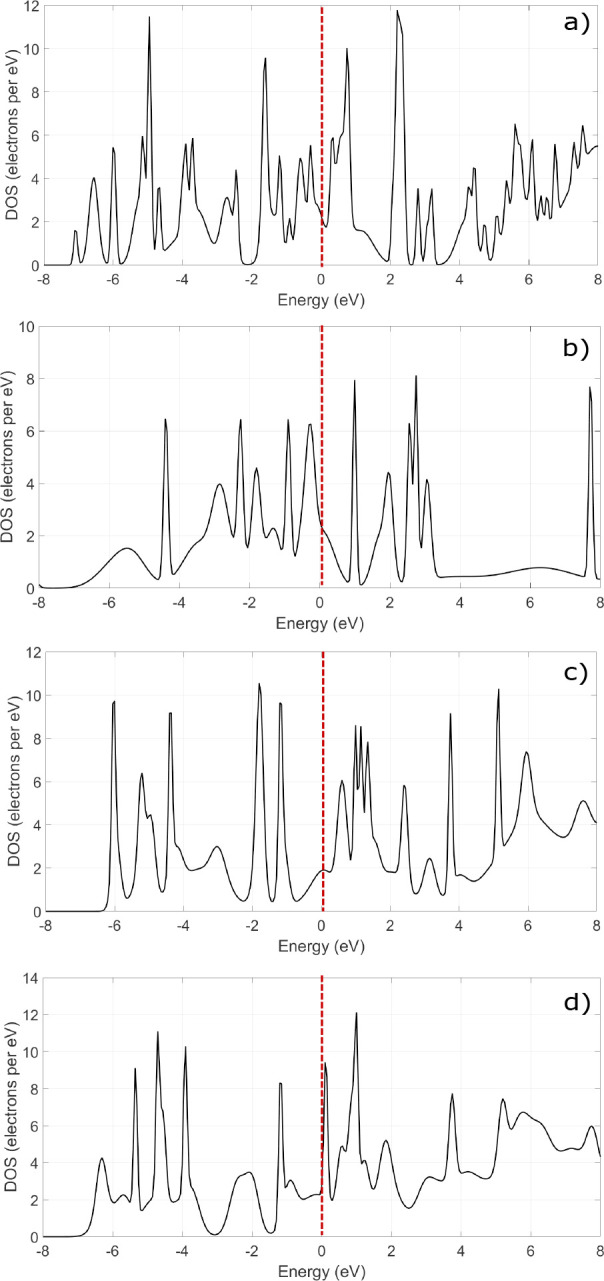
Calculated DOS for (a) Mo_2_CS_2_, (b) Mo_2_CS_2_–Li, (c) MoVCS_2_, (d) MoVCS_2_–Li. The Fermi level is shifted at 0 eV (red dashed line).

Finally, in Figure 5, we see the calculated DOS for each structure. All materials are metallic (no bandgap at 0 eV, i.e., the Fermi level), a property that is essential for electrodes in Li-ion batteries.

## Conclusions

Rechargeable ion batteries are energy storage devices whose operation is based predominantly on the intercalation of ions.^43^ In general, an ion battery consists of a cathode (positive electrode) and an anode (negative electrode) in contact with an electrolyte which contains ions. The two electrodes are separated by a microporous polymer membrane (separator) which stops the electrons from passing between them alongside the ions.^44^

During charging of an ion battery cell, ions leave the positive electrode and move through the electrolyte to the negative electrode. We have, therefore, a storage of energy to the anode. During discharging of the battery, this energy is released, powering the battery, and the ions move back to the cathode.

One of the most active research fields regarding Li-ion batteries is the increase of their rate performance in order to reduce charging time which is important to the electric vehicles’ market.^45^ The materials of the electrodes are crucial for the performance of Li-ion batteries, as they determine capacity, cell voltage, and cyclability.^44^

**Figure 6:**
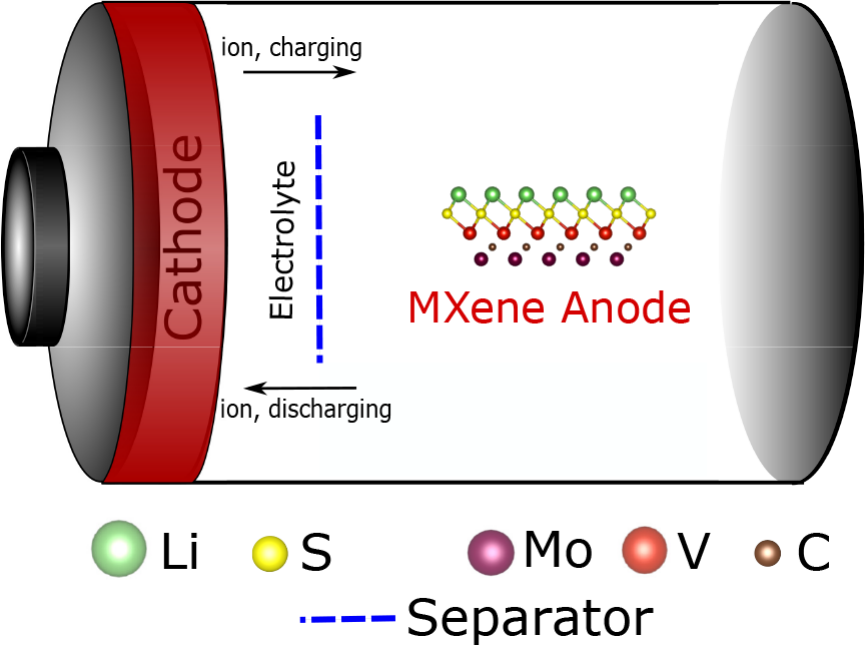
Inner workings of an ion battery with a MoVCS_2_ MXene anode.

Anode materials are still predominantly carbon-based; however, these anodes have almost reached their theoretical maximum capacity (372 mAhg^–1^^44^). For this reason, carbon alternatives are being sought, with a particular focus on MXenes. A schematic of the working cycle of an ion battery, including an MoVCS_2_ MXene anode, for example, can be seen in Figure 6.

In the present study, we examined the use of the Mo_2_CS_2_ MXene as an anode material for Li-ion batteries, where the upper layer of Mo atoms has been substituted by V. We found that this substitution renders the initial Mo_2_CS_2_ more thermodynamically stable, although experimental work has not yet been applied in depth to the kind of materials like the MoVCS_2_ MXene. Furthermore, the substitution of the entire top Mo layer with V was shown to improve the mobility of the Li ion by 95%, while the material retains its metallic character. This fact makes the MoVCS_2_ material a promising candidate for the negative electrode in Li-ion batteries since it allows for faster charge/discharge rates than the Mo_2_CS_2_ MXene.
